# Polyelectrolyte Multicomponent Colloidosomes Loaded with Nisin Z for Enhanced Antimicrobial Activity against Foodborne Resistant Pathogens

**DOI:** 10.3389/fmicb.2017.02700

**Published:** 2018-01-15

**Authors:** Taskeen Niaz, Saima Shabbir, Tayyaba Noor, Rashda Abbasi, Zulfiqar A. Raza, Muhammad Imran

**Affiliations:** ^1^Department of Biosciences, COMSATS Institute of Information Technology, Islamabad, Pakistan; ^2^Department of Materials Science and Engineering, Institute of Space Technology, Islamabad, Pakistan; ^3^School of Chemical and Materials Engineering, National University of Sciences and Technology, Islamabad, Pakistan; ^4^Cancer Research, Institute of Biomedical and Genetic Engineering, Islamabad, Pakistan; ^5^Department of Applied Sciences, National Textile University, Faisalabad, Pakistan

**Keywords:** bacteriocin, chitosan, chitosomes, food-grade delivery system, MDR pathogens, nano-liposome, *Listeria monocytogenes*

## Abstract

Food grade micro- or nano-carrier systems (NCS) are being developed to improve the controlled release of antimicrobial agents. To augment the stability of liposomal NCS and to overcome the limitations associated with the use of free bacteriocin (nisin) in the food system, multi-component colloidosomes (MCCS) were developed by electrostatic interactions between anionic alginate and cationic chitosan (multilayer) around phospholipids based liposomes (core). Zeta-sizer results revealed the average diameter of 145 ± 2 nm, 596 ± 3 nm, and 643 ± 5 nm for nano-liposome (NL), chitosomes (chitosan coated NL) and MCCS, respectively. Zeta potential values of NCS varied from −4.37 ± 0.16 mV to 33.3 ± 6 mV, thus both chitosomes (CS) and MCCS were positively charged. Microstructure analysis by scanning electron microscope (SEM) revealed relatively higher size of MCCS with smooth and round morphology. TGA and DSC based experiments revealed that MCCS were thermally more stable than uncoated liposomes. Encapsulation efficiency of nisin in MCCS was observed to be 82.9 ± 4.1%, which was significantly higher than NL (56.5 ± 2.5%). FTIR analyses confirmed the cross-linking between sodium alginate and chitosan layer. Both qualitative (growth kinetics) and quantitative (colony forming unit) antimicrobial assays revealed that nisin loaded MCCS have superior potential to control resistant foodborne pathogens including *Staphylococcus aureus, Listeria monocytogenes*, and *Enterococcus faecalis*, (5.8, 5.4, and 6.1 Log CFUmL^−1^ reduction, respectively) as compared to free nisin, loaded NL or CS. Controlled release kinetics data fitted with Korsmeyer–Peppas model suggested that nisin release from MCCS followed Fickian diffusion. Cytotoxic studies on human blood cells and HepG2 cell lines revealed hemocompatibility and non-toxicity of MCCS. Thus, due to enhanced controlled release, stability and biocompatibility; these multi-component colloidosomes can be useful for incorporating antimicrobial agents into functional foods, beverages and pharmaceutical products to combat pathogenic and spoilage bacteria.

## Introduction

Food spoilage caused by pathogenic microorganisms during food storage and distribution has serious impact on food quality, shelf-life and food loss (Lopes and Brandelli, 2017). Antimicrobial polypeptides such as nisin are purposefully added in the food systems to control the microbial growth. Nisin, only bacteriocin approved by Food and Drug Authority (Generally Recognized as Safe) and European Union (E234), is known to exert inhibitory effect toward Gram-positive foodborne pathogens e.g., *Listeria monocytogenes, Staphylococcus aureus*, and *Enterococcus faecalis*. Directly added bacteriocin to food products may face many challenges e.g., loss of antimicrobial activity, limited stability against chemical or physical degradation, interactions with different food components, its degradation and electrostatic repulsion, uncontrolled release and possible adverse effects on physical qualities of food structure (Fu et al., 2016). One approach to address these problems is the use of food grade nano-carrier systems to improve the stability, bioavailability and activity of antimicrobial agents (Khan and Oh, 2016).

Liposomes are attractive nano-delivery systems that provide number of benefits including large scale production, enhanced stability of encapsulated agents against enzymatic and chemical degradation during food processing. Liposomes have been widely used in food industries because of their biocompatibility, biodegradability, non-toxicity and small size (Imran et al., 2015; Emami et al., 2016; Alavi et al., 2017). However, quick oxidation during storage and lack of stability of liposomal membrane, which results in burst release of active agent, are major obstacles for liposomes' application in the food industry (Liu et al., 2016). In this context, surface modification of liposomes has been investigated to enhance their stability and functionality (Nguyen et al., 2016). For example, coating liposomes with biopolymer can improve its physico-chemical stability, increase drug pay load and enhance its *in-vitro* digestibility in biologically relevant media (Liu et al., 2017).

Previous studies have revealed that decoration of liposomal surface with biomacromolecules is an effective way to reduce the damage of lipid membrane and associated leakage of encapsulated compounds (Caddeo et al., 2016; Tan et al., 2016; Adamczak et al., 2017; Peng et al., 2017). Chitosan (CTS) is a carbohydrate based poly-cationic linear polymer consisting of (1–4) linkages between D-glucosamine and N-acetyl-D-glucosamine units, and it has demonstrated high biodegradability and biocompatibility (Niaz et al., 2016). Due to the presence of amino groups, chitosan acts as a cationic polymer and makes electrolytic complexes with the oppositely charged molecules (Paques et al., 2014; Li et al., 2016). Liposomes coated with CTS can be prepared through ionic interaction between liposomes and an oppositely charged chitosan. A recent study has highlighted the potential use of CTS as a stability-enhancing agent and bio-adhesive material by coating on the liposomal surface (Pistone et al., 2017). However, one of the major limitation of CTS as a coating agent on phospholipids is its easy dissolution in low pH, which results in subsequent release and denaturation of encapsulated agent (Raval et al., 2017).

Sodium alginate (ALG), a water-soluble anionic polymer, is used extensively as a thickener, emulsifier, and stabilizer in the food and pharmaceutical industries (Draget et al., 2016; Tavernier et al., 2016; Khalil et al., 2017). Though ALG can be layered on the external membrane of liposomes for stability purposes, its dissolution in high-pH conditions could result in loss of its stabilizing effect on liposomes and release of entrapped contents (Manatunga et al., 2017). Thus, polyelectrolyte complexation of both CTS and ALG can display improved dissolution of CTS and ALG in different pH ranges of the food system, as reported previously for inuline (Gandomi et al., 2016).

Thus, the aim of the present study was to fabricate, characterize and evaluate nisin loaded polyelectrolyte multi-component colloidosomes (MCCS) with layer by layer (LbL) self-assembly of CTS and alginate on nano-liposomes. Chitosan was self-assembled on the surface of cationic nanoliposomes prepared by ultrasonication process, and negatively charged alginate was then deposited on the outer layer of cationic chitosomes (CS). Chitosan–alginate stabilized delivery systems were then characterized by using scanning electron microscopy (SEM), Fourier transform infrared spectroscopy (FTIR), thermal gravimetric analysis (TGA) and differential scanning calorimetry (DSC). Bacteriocin loaded MCCS were also evaluated for their antimicrobial potential against milkborne resistant pathogens e.g., *Listeria monocytogenes, Staphylococcus aureus* and *Enterococcus faecalis*. Furthermore, *in vitro* nisin release kinetics from these formulations were also studied at different pH and temperatures to assess the sustained release of active agent.

## Materials and methods

### Materials

Sodium alginate [molecular formula: (C_6_H_7_NaO_6_)_n_, molecular weight: 176.10 g/mol] was purchased from VWR international. Calcium chloride (CaCl_2_) was purchased from SERVA electrophoresis GmbH. Chitosan (medium molecular weight) 85% degree of deacetylation, acetic acid (glacial, ≥99.85%), sodium dodecyl sulfate (SDS) and galactose were purchased from Sigma-Aldrich USA. Growth medium e.g., Muller Hinton, Brain heart infusion and Nutrient agar for strains' growth and preservation were also purchased from Sigma-Aldrich. RapID ONE System kits for bacterial identification were procured from Thermo Fisher Scientific. Nisin Z (90% pure) was purchased from Honghao Chemical Co. Shanghai, China. Purified soy lecithin with ⩾94% phosphatidylcholine, was provided by Lipoid (Ludwigshafen, Germany). Sodium pyruvate, glutamine, hepes, penicillin/streptomycin and Dulbecco's Modified Eagle Medium (DMEM) for hepG2 cell lines were purchased from Invitrogen.

### Isolation, identification, and resistance profiling of milkborne pathogens

Gram-positive foodborne pathogens e.g., *Listeria monocytogenes, Staphylococcus aureus*, and *Enterococcus faecalis* were collected from different milk industries in Pakistan. Samples were serially diluted and spread on different differential growth media to isolate desired pathogenic strains, and then morphologically selected colonies were individually purified and subjected for identification. All isolates were examined for biochemical properties using RapID ONE System kits (Thermo Fisher Scientific). Multi drug resistant (MDR) profiles of the identified strains were carried out by disc diffusion method (Benmansour et al., 2016).

### Quantification of Nisin by nano-photometer

Stock solution of nisin (1 mg/mL) was prepared by dissolving 0.01 g of nisin powder in 10 mL of distilled water. Working solutions were made by various serial dilutions from stock solution. Wavelength selected after wave scan was 291 nm by using Multiskan™ GO Microplate Spectrophotometer. Optical density (OD) values were recorded for each dilution in triplicate and then respective standard curve was prepared by Bradford assay.

### Minimum inhibitory concentration (MIC) of Nisin Z

MIC of bacteriocin was obtained by spot on lawn method. Nisin solutions at different concentrations (1,000–5 μg/mL) were made by serially diluting the stock solution. Freshly cultured colony of each pathogen was spread on nutrient agar plate separately. Afterwards, 10 μL of sample was taken from each nisin dilution and spotted it on freshly spread bacterial culture. MIC was the lowest concentration of bacteriocin resulting in inhibition of visible growth after 24 h (Balay et al., 2017).

### Optimization of multicomponent colloidosomes (MCCS)

Solution A was prepared by adding soy lecithin (10 mg/mL) in distilled water and stirred for 4 h (1,200 rpm). After complete dissolution of lecithin dispersion, it was sonicated for 15 min by ultra-sonicater. Solution B was prepared by dissolving CTS (0.6% w/v) in 1% (v/v) glacial acetic acid aqueous solution, and solution C was prepared by adding 0.5% w/v sodium alginate in dH_2_O. Afterwards, pH of both solution B and C were adjusted to 5 followed by overnight stirring at 1,200 rpm and filtration. Chitosan coated nano-liposomes i.e., chitosomes (CS) were prepared by adding equal volume of solution A dropwise into solution B (1:1, v/v), and then incubated for 1 h under gentle stirring. Dispersion was sonicated for 15 min after incubation. Multicomponent nanoliposomes (LPs-CTS-ALG) were prepared by ionotropic pre-gelation methods. For the fabrication of MCCS equal volume of chitosomes were added into the solution C (1:1, v/v) and incubated for 1 h under constant magnetic stirring. Subsequently, equal volume of calcium chloride solution (18 mM of CaCl_2_) was added in the above solution. Afterwards, the solution was subjected to sonication for 15 min to obtain multi-component colloidosomes (MCCS). Both CS and MCCS were centrifuge at 15,000 g for 30 min for further characterization of EE. Nisin loaded carrier systems were fabricated accordingly by dissolving nisin in distilled water before soy lecithin addition in solution A.

### Size and zeta potential of void and nisin-loaded carrier systems

Mean particle size and zeta potential of the blank and bacteriocin loaded MCCS were calculated by using Zeta-sizer Nano-ZS (Malvern, UK). This instrument works on the principle of dynamic light scattering (DLS). It comprises of 4 mW He/Ne laser, photo multiplier, measurement cell and correlator. Briefly, 3 mL suspension of nanoliposomes (LPs), CTS coated liposomes and MCCS (LPs-CTS-ALG) were diluted with deionized water and average size distributions and zeta potential of particles were measured at 25°C with refractive index of 1.33.

### Physical stability analysis of nano-formulations with DLS

DLS technique was used to determine the average size, zeta potential (ZP) and polydispersity index (PDI), which are considered as stability indicators for nano-carrier systems. PDI illustrates the homogeneity of the colloidal dispersion and zeta potential determines the net charge on NCS, both of these parameters help to predict the stability of colloidosomes during storage period (Koirala et al., 2016). Therefore, to determine the stability of MCCS, mean particle size, polydispersity index (PDI) and zeta potential (ZP) were measured for 3 weeks at room temperature (25°C). All the measurements were taken in triplicate.

### Encapsulation efficiency of nisin

MCCS dispersion was centrifuge at 15,000 rpm and un-encapsulated (free) nisin in the supernatant was measured by checking its optical density (OD) value at 291 nm with nano-spectrophotometer (Implen). By comparing OD values of nisin with the standard curve, specific concentration of the encapsulated bacteriocin was calculated. The total amount of nisin was referred to its initial concentration added into the formulation during NCS fabrication. Encapsulation efficiency (EE %) of bacteriocin was calculated using the Equation (1).

(1) $$\begin{array}{l} \text{EE}\%=[\text{Encapsulated drug }(\text{Total drug }-\text{ Free drug})]\\ \text{ /}[\text{Total drug}]\;\times \text{100} \end{array}$$

### Scanning electron microscopy (SEM)

To investigate the morphology of void and nisin loaded multicomponent NCS, SEM was performed by using Field Emission Scanning Electron Microscope (FE-SEM) (Tescan, USA). About 2–5 μL of NCS suspension was spread on a glass slide (1 × 1 cm) and dried at room temperature. Air-dried samples were sputter coated with carbon. Samples were analyzed for shape and size using an electron acceleration voltage of 5–10 KeV (Sadiq et al., 2016).

### Thermal analysis of biopolymer, active agent and nano-antimicrobials

#### Thermogravimetric analysis (TGA)

TGA analysis was performed on Shimadzu TGA-50, Japan. Temperature scans of the samples were carried out under dynamic nitrogen atmosphere with heating rate of 10°C/min. Minimum 10 mg sample was placed in alumina crucible pan and subjected to TGA analysis with temperature range from 0 to 600°C (Sood et al., 2017).

#### Differential scanning calorimetry (DSC)

DSC studies were performed using a Shimadzu DSC-50 equipped with a Mettler Toledo DSC 1 Stare System. Aliquots of about 10 mg of each sample were placed in an aluminum pan before performing DSC measurements. Then, each calorimetric pan was sealed and submitted to DSC analysis for heating range from 0 to 300°C, at the rate of 2°C/min, and 10°C/min under a nitrogen flow of 20 cm^3^/min. Reproducibility was checked by analyzing the samples in triplicate.

#### Fourier transformed infrared spectroscopy (FTIR)

FTIR based spectroscopic investigation was performed using a PerkinElmer 100 FTIR spectrometer (PerkinElmer, Italy). FTIR spectra of powdered nisin and polymer were recorded with KBr disc method as reported earlier (Niaz et al., 2016) with few modifications. Powder/dried samples were mixed with an appropriate amount of KBr to obtain a final concentration (1% w/w) of samples, whereas liquid nano-formulations were directly analyzed on FTIR spectrometer. FTIR transmission spectra of empty multicomponent NCS and nisin loaded NCS were recorded from 500 to 4,000 cm^−1^ with a resolution of 1 cm^−1^.

### Antimicrobial potential of nano-antimicrobials

#### Antimicrobial potential of different nano-active formulations on inoculated agar media

To find out the antibacterial activity of MCCS, 10 uL of nano-formulation was placed on nutrient agar medium inoculated with *Listeria monocytogenes, Staphylococcus aureus*, and *Enterococcus faecalis* separately on individual Petri dish. Plates were refrigerated at 4°C for 2 h to allow the diffusion of bacteriocin loaded NCS in the agar medium without microbial growth and then incubated at 37°C.

#### Growth kinetics of foodborne pathogens exposed to nano-antimicrobials

The growth kinetics of foodborn pathogenic strains were tested by exposing them to nano-antimicrobials by using a modified protocol reported earlier (Sadiq et al., 2016). Optical density value of inoculated broth containing either free or encapsulated bactericidal agent were recorded at 595 nm in 96 well plate by using micro-plate absorbance reader (Bio-Rad iMark).

#### Quantitative analysis of nano-antimicrobials' potential: colony forming unit (CFUml^−1^)

To determine microbial viability after exposure with nano-antimicrobials, CFU analyses were performed by counting the number of colonies on agar plate as described previously (Jamil et al., 2016b). Briefly colony forming unit (CFU) measurements were performed by taking 100 μL of sample from each tube (inoculated with pathogens and with different nano-formulations e.g., NL, CS and MCCS) and serially diluted up to 10^8^ dilution factors. After specific intervals, 20 μL from each diluent was spread on LB agar with a sterile cotton swab and Petri plates were incubated for 24 h. Individual bacterial colonies on the agar plate were counted manually.

#### *in vitro* controlled release kinetics of bacteriocin

*In vitro* controlled release of nisin from polyelectrolyte multicomponent NCS was performed as described previously by Niaz et al. (2016), with slight modifications. Nisin loaded NCS were incubated in 0.1 M phosphate buffer saline (PBS) with pH 7 and pH 4. Controlled release profiles were observed at two different temperatures i.e., 4 and 37°C, respectively. After regular intervals, 2 mL suspension (nisin loaded MCCS in PBS) was withdrawn, centrifuged and analyzed for free nisin by nano-photometer. To determine the release kinetics of nisin from NCS Korsmeyer–Peppas and Higuchi mathematical models were applied.

Higuchi model (Equation 2), was used to determine the release of drug by diffusion, which plots percentage drug release against the square root of time. Where K_H_ is Higuchi dissolution constant and Q is the amount of active agent release in time “t” (Jain and Jain, 2016).

(2) $$\text{Q}={\text{K}}_{\text{H}}{\text{t}}^{1/2}$$

Moreover, Korsmeyer–Peppas model (Equation 3) was applied to assess Fickian as well as non-Fickian, anomalous, case II and super case II transport of encapsulated antimicrobial from MCCS at different pH and temperatures. Initial 60% drug release data was fitted with this model (Hervault et al., 2016).

(3) $${\text{Mt/M}}_{\infty }={\text{K}}_{\text{kp}}{\text{t}}^{\text{n}}$$

Where Mt is the mass of bacteriocin (nisin) released at time t, M∞ represents the total mass of nisin to be released and k is a constant which represents the structural characteristics of the nano-carrier systems. The exponent n indicates the type of release and its value changes with the change in release mechanism of diffusion e.g., nisin diffusion will be Fickian when *n* = 0.43. For NCS having high polydispersity, value of n less than 0.43 could also be possible and considered as Fickian diffusion (Koirala et al., 2016). The values of n between 0.5 and 1.0 are indicative of anomalous, Case II transport, super-case II non-Fickian diffusion (de Oliveira Pedro et al., 2018).

#### *in vitro* hemolysis analysis

*In vitro* hemolysis assay of NCS was performed as described previously (Jamil et al., 2016a). Briefly human whole blood sample was collected in Li–heparin as anti-coagulant vacutainer. Blood sample was incubated with 1% of nano-antimicrobials' solution at 37°C for 45 min. Unexposed samples were taken as negative control, whereas sodium dodecyl sulfate (1% SDS) solution was added as positive control in the blood sample. After incubation, samples were centrifuged at 14,000 rpm for 5 min. After centrifugation, supernatant was collected from each samples and OD was measured at 540 nm by Multiskan™ GO microplate spectrophotometer. Percentage hemolysis was calculated relative to the untreated control. All assay values were observed in duplicate.

#### Cytotoxicity assessment on human HepG2 cell line

HepG2 cells were cultured in DMEM with 10% (v/v) heat-inactivated fetal bovine serum (FBS), Na-pyruvate (1 mM), l-glutamine (2 mM), galactose (10 mM), hepes (5 mM), and penicillin/streptomycin (100 iU/mL). The cells were kept at 37°C under humidified condition with 5% carbon dioxide (CO_2_) and 95% air.

The sulforhodamine B (SRB) cell viability assay was used to detect the cytotoxicity of the nanocarrier systems against HepG2 (ATCC HB-8065TM) cell lines as described previously (Dai et al., 2017). Briefly, HepG2 cells were harvested overnight in 96-wells plates (15,000 cells/well), Next day, cells were exposed to nano-antimicrobials and incubated for 24 h at 37°C with 5% CO_2_. After incubation, the monolayers of cells were fixed with 10% (w/v) trichloro acetic acid (TCA) and kept at 4°C for at least 2 h.

The samples were carefully washed with deionized water, air dried and stained for 30 min with 0.4% SRB solution. Samples were washed with 1% acetic acid and dried overnight. Photographs of the dried samples were taken by Olympus IMT-2 inverted microscope equipped with digital camera. The incorporated dye was solubilized in 0.01M Tris solution and OD at 565 nm was measured on spectrophotometer (de Oliveira Pedro et al., 2018). Unexposed cells were considered as control and relative cell viability was calculated as a percentage in triplicate. The viability of the control cells was considered as 100%. Relative % cell viability were calculated by Equation (4).

(4) $$\begin{array}{l} \text{Relative viability}=[\text{OD of test sample }-\text{ OD sample control}\\ \text{ /OD unexposed sample }-\text{ OD Media}]\times \text{100} \end{array}$$

## Results and discussion

### Resistance and susceptibility profiling of pathogens

**Figure 1:**
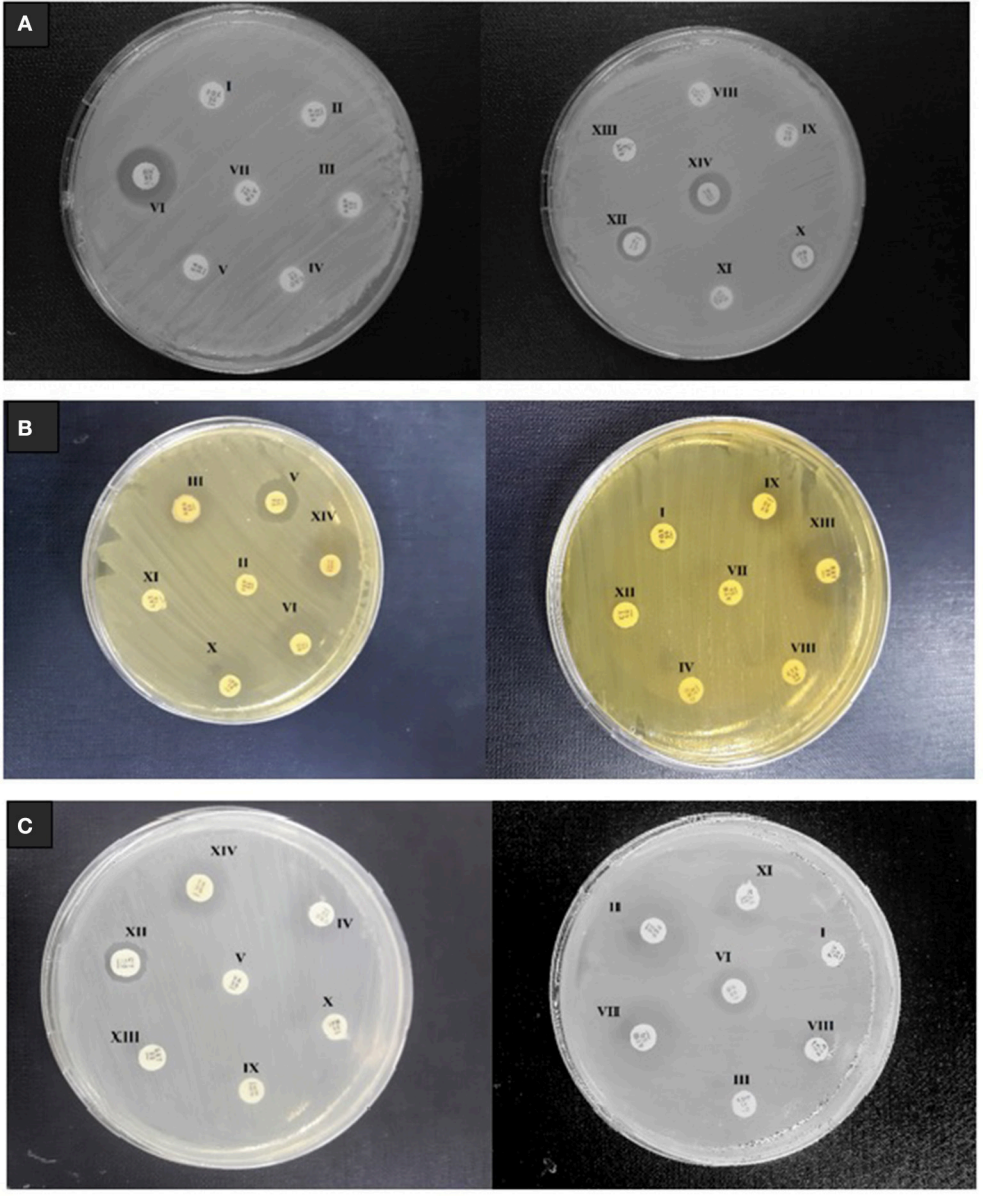
Multi drug resistance profiling of foodborne pathogens isolated from milk samples **(A)** *E. faecalis* **(B)** *L. monocytogenes*, and **(C)** *S. aureus*. The tested discs containing different antibiotics include FOX (I) FEP (II) AMP (III) CRO (IV) VA (V) MH (VI) ATM (VII) CTX (VIII) KF (IX) IPM (X) CAZ (XI) CT (XII) SXT (XIII) TE (XIV).

To assess multidrug resistance profiling; *Enterococcus faecalis, Listeria monocytogenes* and *Staphylococcus aureus* were tested against 14 different commercially available antibiotics and zone of inhibition of each antibiotic was measured (Figure 1). To categorize the pathogens as susceptible (S), Intermediate (I), or resistant (R); results were compared to CLSI guidelines (Table 1). *E. faecalis, Listeria monocytogenes* and *Staphylococcus aureus* were completely resistant against 1st and 3rd generation cephalosporins e.g., cefazolin (KF) and cefotaxime (CTX). All these pathogens were resistant to vancomycin (VA), so we can mark them as vancomycin resistant strains of *enterococcus* (VRE)*, Listeria and Staphylococcus* (VRSA). The vanA and vanB genes involved in the regulation and expression of vancomycin resistance, which often reside on a plasmid, are responsible for emerging vancomycin resistance in *E. faecalis* (Greninger et al., 2016). In addition to the resistance against vancomycin, *S. aureus* was also resistant to cefoxitin (FOX), hence considered as methicillin resistant *S. aureus* (MRSA). Due to horizontal transfer of resistance genes present on the plasmid; S*taphylococcus* spp., *E. faecalis* and *Listeria monocytogenes* exhibited complete resistance against β-lactam antibiotic i.e., ampicillin (AMP). All pathogens demonstrated resistance against carbapenems. Multidrug resistant including vancomycin and methicillin resistant foodborne pathogens are considered as health threat of the twenty first century (Miller et al., 2016). To treat emerging antibiotic-resistance in foodborne pathogens, natural antimicrobial peptides have been suggested as a good alternative for conventional antibiotics. Among all the antimicrobial peptides (AMPs), nisin is the only class 1 bacteriocin (lanthionine-containing antibiotics) approve by FDA to be utilized as food preservatives. As a primary test for antibacterial activity, free nisin (non-encapsulated) revealed MIC value of 10, 50, and 200 μg/mL against *Staphylococcus aureus, Listeria monocytogenes* and *Enterococcus faecalis*, respectively. Nisin acts on bacteria by binding to lipid II (inhibit cell wall synthesis) and by pore formation on cell membrane of the targeted pathogens (Han et al., 2017; Jorge et al., 2017)

Table 1Resistance profiling of foodborne pathogens against tested antibiotics.

**Table:** 

**Antimicrobial agent**	**Disc content (μg)**	**Inhibition zone diameter (mm)**		
		**S (Sensitive)**	**I (Intermediate)**	**R (Resistant)**
Cefoxitin (FOX)	30	≥23	–	–
Cefepime (FEP)	30	≥18	15–17	≤ 14
Ampicillin (AMP)/Amoxicillin (AMC)	10/30	≥22	19–21	≤ 18
Ceftriaxone (CRO)	30	≥26	–	–
Vancomycin (VA)	30	≥17	15–16	≤ 14
Minocycline (MH)	30	≥19	15–18	≤ 14
Aztreonam (ATM)	30	≥26	18–20	≤ 17
Cefotaxime (CTX)	30	≥26	–	–
Cephalothin (KF)	30	≥15	–	–
Imipenem (IPM)	10	≥19	16–18	≤ 15
Ceftazidime (CAZ)	30	≥18	15–17	≤ 14
Colistin sulfate (CT)	10	≥17	10–16	≤ 11
Sulphamthoxazole/trimethoprim (SXT)	1.25/23.75	≥16	11–15	≤ 10
Tetracycline (TE)	30	≥19	15–18	≤ 14

**Table:** 

**Pathogens**	**FOX I**	**FEP II**	**AMP III**	**CRO IV**	**VA V**	**MH VI**	**ATM VII**	**CTX VIII**	**KF IX**	**IPM X**	**CAZ XI**	**CT XII**	**SXT XIII**	**TE XIV**
**ZONE OF INHIBITION (MM)**														
*E. faecalis*	–	–	–	–	–	18	–	–	–	–	–	10	–	12
*L. monocytogenes*	–	25	–	24	10	14	22	–	–	13	20	24	17	22
*S. aureus*	–	12	–	–	–	10	13	–	–	–	12	10	–	11

*Disk diffusion quality control range for pathogens according to CSLI guidelines 2017*.

### Characterization of size and surface charge of nano-antimicrobials

Dynamic light scattering (DLS) based assays revealed that the particle size and zeta potential of empty nano-liposomes were 145 ± 2 nm and −4.37 ± 0.16 mV, respectively, with a PDI of 0.21. After surface engineering with layers of CTS and ALG, the size increased to 596 ± 3 nm and 643 ± 5 with ζ potential values varied from 16.5 ± 5.8 mV and 33.3 ± 6.07 mV for chitosomes and multi-component colloidosomes, respectively (Table 2).

**Table 2:** Average size, PDI (polydispersity index), ζ potential and encapsulation efficiency of empty and nisin loaded liposomes, chitosan coated liposomes (chitosomes) and multicomponent colloidosomes with standard deviation values (*n* = 3).

**Nano formulations**	**Size nm ± SD**	**PDI**	**ζ potential (mV ± SD)**	**EE%**
Void liposomes	145 ± 2	0.21	−4.37 ± 0.16	–
Void chitosomes	596 ± 3	0.24	16.5 ± 5.80	–
Void multicomponent colloidosomes	643 ± 5	0.19	33.3 ± 6.07	–
Nisin-liposome	161 ± 2	0.15	−3.47 ± 0.13	56.53 ± 2.5%
Nisin-chitosomes	768 ± 5	0.24	33.0 ± 5.21	82.43 ± 1.5%
Nisin-multicomponent colloidosomes	844 ± 6	0.25	33.3 ± 4.07	83.35 ± 2.2%

After encapsulation with nisin, changes in nanoparticle size were observed in NLs as well as in chitosan coated liposomes and MCCS. Average size of MCCS was increased to 844 ± 6 nm after nisin encapsulation. Similarly, particle size of nanoliposmes varied from 145 to 161 nm, which confirmed the encapsulation of nisin inside the lipid bilayer and core. Previously, it was observed that main factors influencing the average size and zeta potential of multi-component systems were pH, Ca^2+^, ALG, and CTS concentration (Li et al., 2013; Almalik et al., 2017). Moreover it was previously observed that more than 0.2% Ca^2+^ ratio in ALG solution increased the particle size (Caetano et al., 2016).

Zeta potential analysis revealed that MCCS were positively charged and zeta potential remain same even after nisin encapsulation. According to EE results, only 1% of nisin was encapsulated between alginate and chitosan coating in MCCS, therefore zeta potential value remained unaffected.

Positive zeta potential value of MCCS could be due to positively charged Ca^2+^ ion and chitosan. At the beginning, colloidal suspension appeared negatively charged due to the adsorption of anionic ALG. However, after addition of CaCl_2_, the value of zeta potential becomes positive (Chandrasekar et al., 2017). Moreover, when Chitosan concentration was over 0.48%, zeta potential varied toward a drastic positive charge because of excessive chitosan and Ca^2+^ cross-linking (Cheng et al., 2015). Most significantly, zeta potential analysis indicated that relatively higher positive charge of MCCS as compared to NLs or chitosomes can not only contribute toward the stability of nano-formulation but also help in the interaction of NCS with negatively charged bacterial pathogens.

### Physical stability analysis

**Figure 2:**
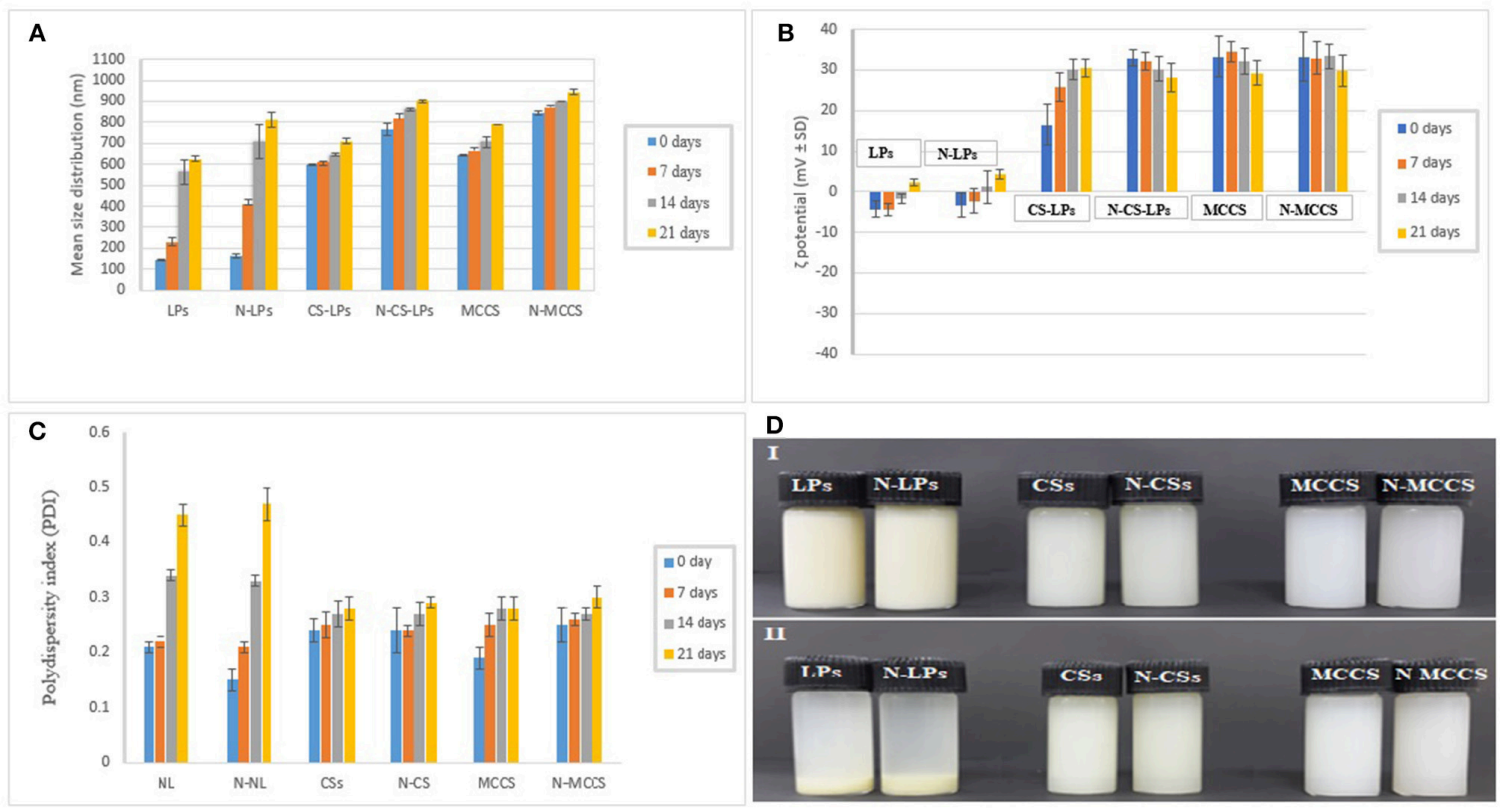
Stability analysis of NLs (Nano liposomes), N-LPs (Nisin loaded liposomes), CSs (blank chitosomes), N-CSs (Nisin loaded chitosomes), MCCS (blank multicomponent colloidosomes) and N-MCCS (nisin loaded multicomponent colloidosomes), over a period of 21 days. Considering the variables: **(A)** size, **(B)** zeta potential, **(C)** PDI, and **(D)** appearance (where 0 refers to day of fabrication, and 1, 2, and 3 refer to time in weeks).

To ensure physical stability of nano-antimicrobials; particle size, PDI and zeta potential analyses were performed (Figure 2). Visual changes in appearance of different nano-formulations (NLs, CS, MCCS) with or without nisin were studied after storage at room temperature for 3 weeks as well (Figure 2D). It was observed that all the nanocarriers systems remained stable during first week as no drastic change in average size was observed after 7 days. However, after 2 and 3 weeks of storage, remarkable increase in size was observed in blank as well as nisin loaded nanoliposomes (145 ± 2 to 811 ± 4.1 and 161 ± 2 to 790 ± 3.9, respectively). On the other hand, CS and MCCS remained stable during this period as size distribution data had not displayed significant variation (Figure 2A).

Polydispersity index (PDI) is an important parameter to determine the size heterogeneity of the dispersions during storage. PDI data of nano-formulations indicated better stability of CS and MCCS than NLs, as PDI was found to be less than 0.3 in both chitosomes and MCCS with or without bacteriocin, thereby signifying the presence of monodispersed NCS (Figure 2C). However, PDI was >0.4 for uncoated nanoliposomes indicating the formation of aggregates during storage. This aggregation or phase separation in NLs was also visually observed after 21 days (Figure 2D).

ZP indirectly determines the stability of nano-colloidal systems. Negative zeta potential of NLs is due to negatively charged phosphate groups in the head group of phospholipids (Bhattarai et al., 2016). The magnitude of the negative zeta potential on NLs decreased with time (Figure 2B), which might be due to release of positively charged nisin in the solution and due to less availability of negatively charged phosphate group after aggregation. No significant variations in ZP were noticed in void and nisin loaded multicomponent colloidosomes which confirmed their physical stability.

### Measurement of encapsulation efficiency (EE %)

Size of the NCS, chemical structure, composition of polymers and active agent are the main factors affecting encapsulation efficiency of any NCS. Smaller the size, lower will be the EE% and vice versa. EE% of nisin loaded multicomponent nanoparticles was 83 ± 2.2%. Thus, the EE% of nisin for MCCS was almost equal to chitosomes (82 ± 1.5%) but relatively higher than nano-liposomes (56 ± 2.5%) (Table 2). Nisin entrapment efficiency depends on the interaction between outer layer of liposome and chitosan; as well as extent of cross linking between alginate, CTS polymer and divalent cation (Ca^+2^). In MCCS, crosslinking between the two biopolymers occurred due to free NH_2_ group of CTS and –COOH or –OH groups of alginate. This crosslinking increases the stability of multicomponent NCS, makes them less porous for the release of nisin. Therefore, free nisin (which was not entrapped in liposomes) was embedded in the three-dimensional polyelectrolyte shell during coating with polymers. Moreover, nisin Z contains terminal amino group of isoleucine and terminal carboxyl group of lysine which possess pKa values of 9.68 and 2.18, respectively. Nisin Z is more soluble at near neutral or acidic pH. At this pH range (2.5–6.5), ionization of the carboxyl group occurs due to which solubility of nisin increases. At basic pH above 7, de-protonation of terminal amino groups decreases the protein solubility and reduces its positive charge. As a result, reduction in electrostatic interaction between nisin and oppositely charge molecules was observed (Gharsallaoui et al., 2016; Khan and Oh, 2016). Secondly, structural modification in nisin Z starts at basic pH and above its isoelectric point (pI, 7). These modifications occur rigorously via intra molecular interactions, which render nisin less available for interactions with polymer. Thus, these modifications result in reduced EE % as well as antimicrobial efficiency of nisin. As the pKa values of chitosan and alginate (6.2–7 and 3.4–4.4, respectively) (Seeli et al., 2016) are existing in the solubility range of nisin, it resulted in better interaction of nisin with these carbohydrate based polymers. The presence of protonated carboxyl groups could promote the electrostatic or hydrophobic interactions of nisin with polymers in the acidic solution, thus increasing its encapsulation efficiency. As previously reported, the reduction in pH (6.6–3.6) demonstrated a positive effect on the amount of nisin entrapped in the polymeric delivery systems (Krivorotova et al., 2016). Therefore, it is concluded that relatively lower pH and hydrophobicity of nisin controlled the interaction of nisin with biopolymer, which is reflected in relatively higher encapsulation efficiency of nisin in MCCS.

### Scanning electron microscopy (SEM) of multilayer colloidosomes

**Figure 3:**
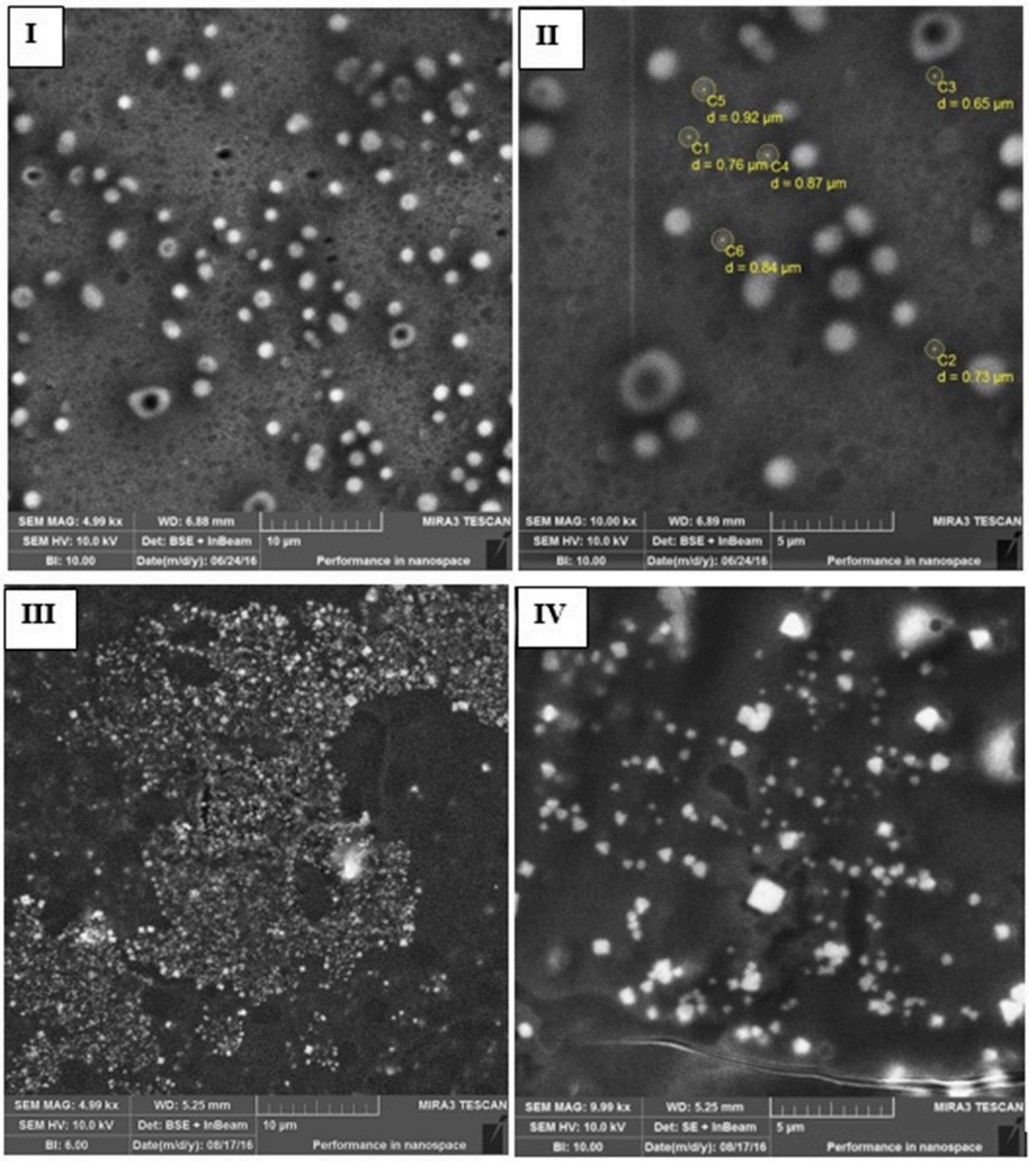
Scanning electron microscopy (SEM) images of blank and nisin encapsulated multicomponent systems including nisin loaded MCCS **(I,II)** and void MCCS **(III,IV)**.

SEM was performed for the morphological characterization of MCCS. Microscopic images revealed that multicomponent NCS with and without nisin were round and smooth in appearance (Figures 3I–IV). Crystalline structures were not observed on the surface of nisin loaded NCS, which indicated that the active peptides were entrapped inside the colloidal system. Size of the multicomponent carrier system ranged from 800 to 900 nm due to multilayer polymer coating on nano-liposomes. Increase in size could be attributed to the gelation and swelling property of alginate during air-drying of samples on glass-slides before carbon coating.

### Differential scanning calorimetry (DSC)

Phase transition and physical aging of nisin and polymer as pure compound and nano-antimicrobial formulations were determined by DSC analyses. In thermogram of sodium alginate, initial endothermic peak was observed at 110°C, whereas higher exothermic peaks were observed at 189°C and 285°C, respectively. Endothermic peaks are correlated with loss of water linked to hydrophilic groups of polymer while exothermic peaks resulted from degradation, dehydration and depolymerization reactions, which are most probably due to partial decarboxylation and oxidation reactions of the –COOH groups present in sodium alginate (El-Houssiny et al., 2016).

**Figure 4:**
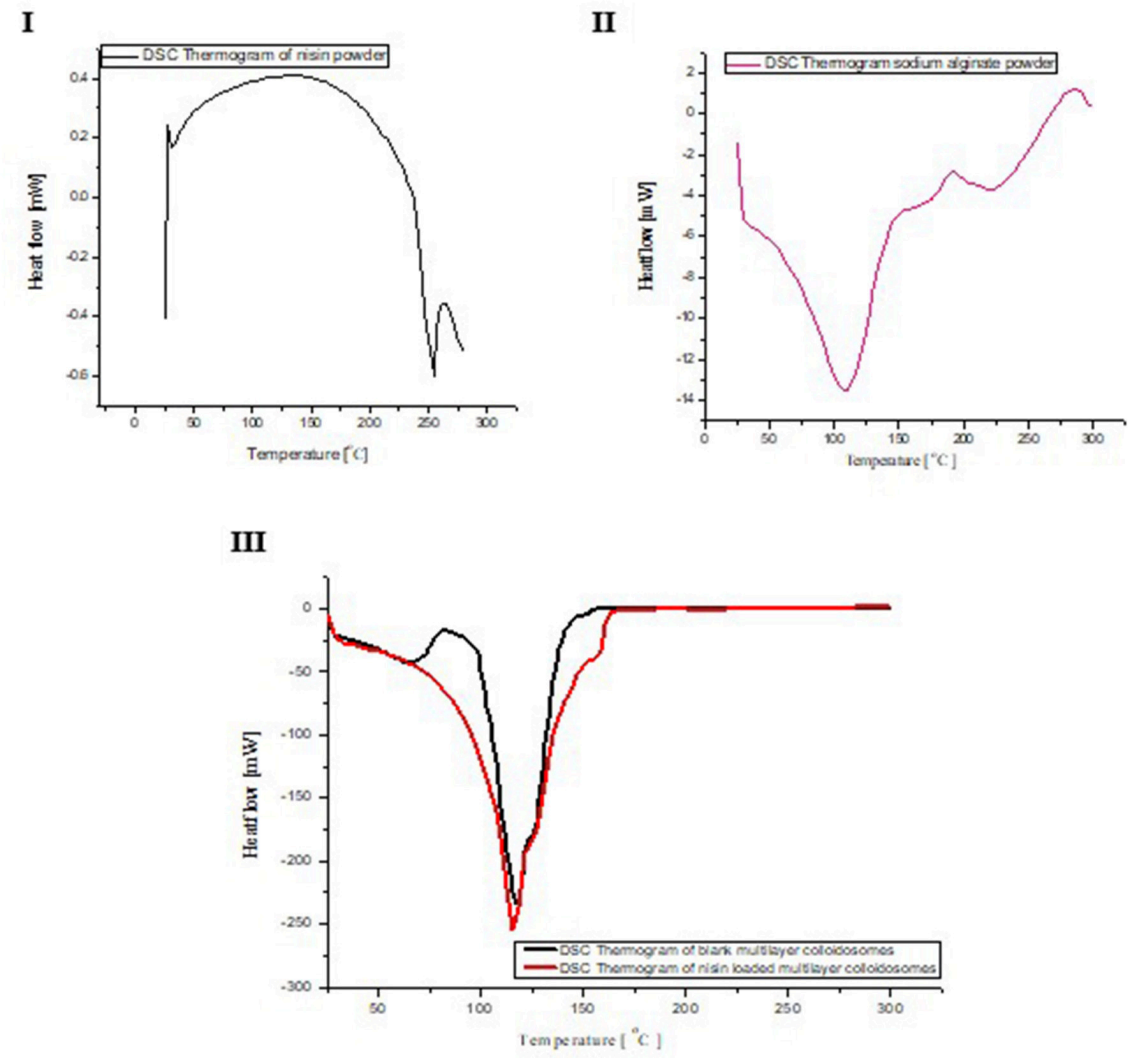
DSC analyses of nisin **(I)**, sodium alginate **(II)**, comparison of DSC curve between void and nisin loaded NCS **(III)**.

Phase transition in empty MCCS was observed from 67 to 98°C which was not present in nisin loaded multicomponent NCS (Figure 4). In DSC analysis of multicomponent NCS, a decrease in melting endotherm (Tm) was observed from 117°C in blank NCS to 115°C in nisin loaded MCCS which could be referred to the cleavage of hydrogen bonds during nisin encapsulation in NCS. Formation of hydrogen bonds in the crystallization phase occurred at 150.5°C. This could be due to degradation of alginate layer at this high temperature, thus exposing the CTS layer. In case of active agent loaded NCS, crystallization endotherm was registered at 158°C which was not very different from blank NCS. This observation confirmed that very low amount of active agent was present between alginate and chitosan layer (Furuya et al., 2017). Once alginate layer degraded at high temperature, it exposed more amorphous content (nisin and chitosan), which increased the temperature value for crystalline endotherm.

### Thermo gravimetric analysis (TGA)

**Figure 5:**
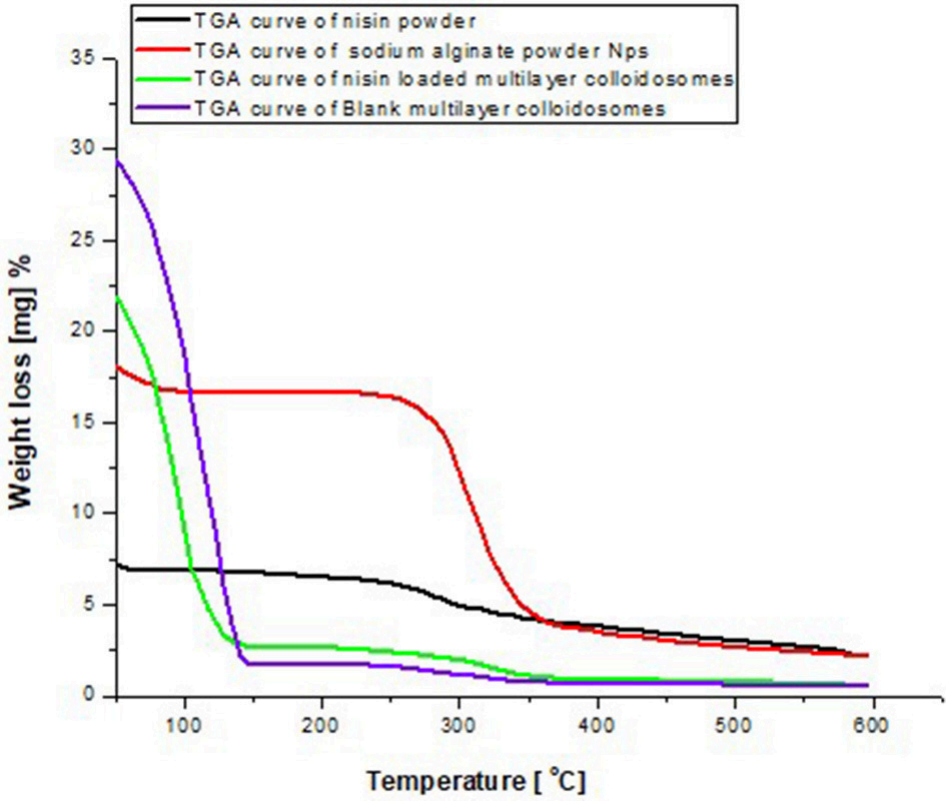
Comparative TGA thermogram between alginate, nisin void and active agent loaded multicomponent NCS.

TGA curves of nisin, alginate and multicomponent colloidosomes are shown in (Figure 5). In TGA analysis of pure nisin, a single major thermal degradation peak was observed at 259°C. However TGA curve of sodium alginate showed three peaks; first peak initiated at 32°C and continued till 92°C corresponding to 1.8% weight loss which can be attributed to the loss of moisture (Pandey and Ramontja, 2016). The second shift in curve appeared at 257°C attributing to the major weight loss of almost 13% due to breaking up of C-H bonds, which refers to the decomposition and degradation of the biopolymer. This decomposition peak initiated from 198°C and continued until 363°C. Furthermore, 2% weight loss was observed during third shift in the TGA peak of ALG, which initiated from 364°C and continued until 591°C. Overall, for sodium alginate powder, the gradual mass loss in TGA curve was observed from 92°C to 363°C.

Similarly, in multilayer NCS, mass loss due to water evaporation was observed in both blank and nisin loaded nanoparticles. Second phase of mass loss was observed before 349°C, which was not observed (negligible) in blank multicomponent nanocarrier systems. No kink or dip for nisin decomposition (259°C) was observed in the TGA curve, which means nisin is not encapsulated between the alginate meshwork, rather it is encapsulated between the core and internal layer of multicomponent colloidosomes. Thus, multilayer coating on nano-liposomes slightly increase the thermal stability, which is in coherence with previous findings for anticancer drug encapsulation system (Manatunga et al., 2017). Insignificant difference was seen in the thermogram of both blank and nisin loaded multicomponent colloidosomes which could be due to the fact that positively charged chitosan and calcium ions can potentially compete with each other for available carboxylic acid sites on the alginate, leaving less pace for nisin to encapsulate in the outer layer of alginate on CS (George and Abraham, 2006). This hypothesis can also be verified from %EE data. Hence nisin is encapsulated either in the core and bilayer of liposomes or in the CTS layer on the surface of nano-liposomes.

### Fourier transformed infrared spectroscopy (FTIR)

**Figure 6:**
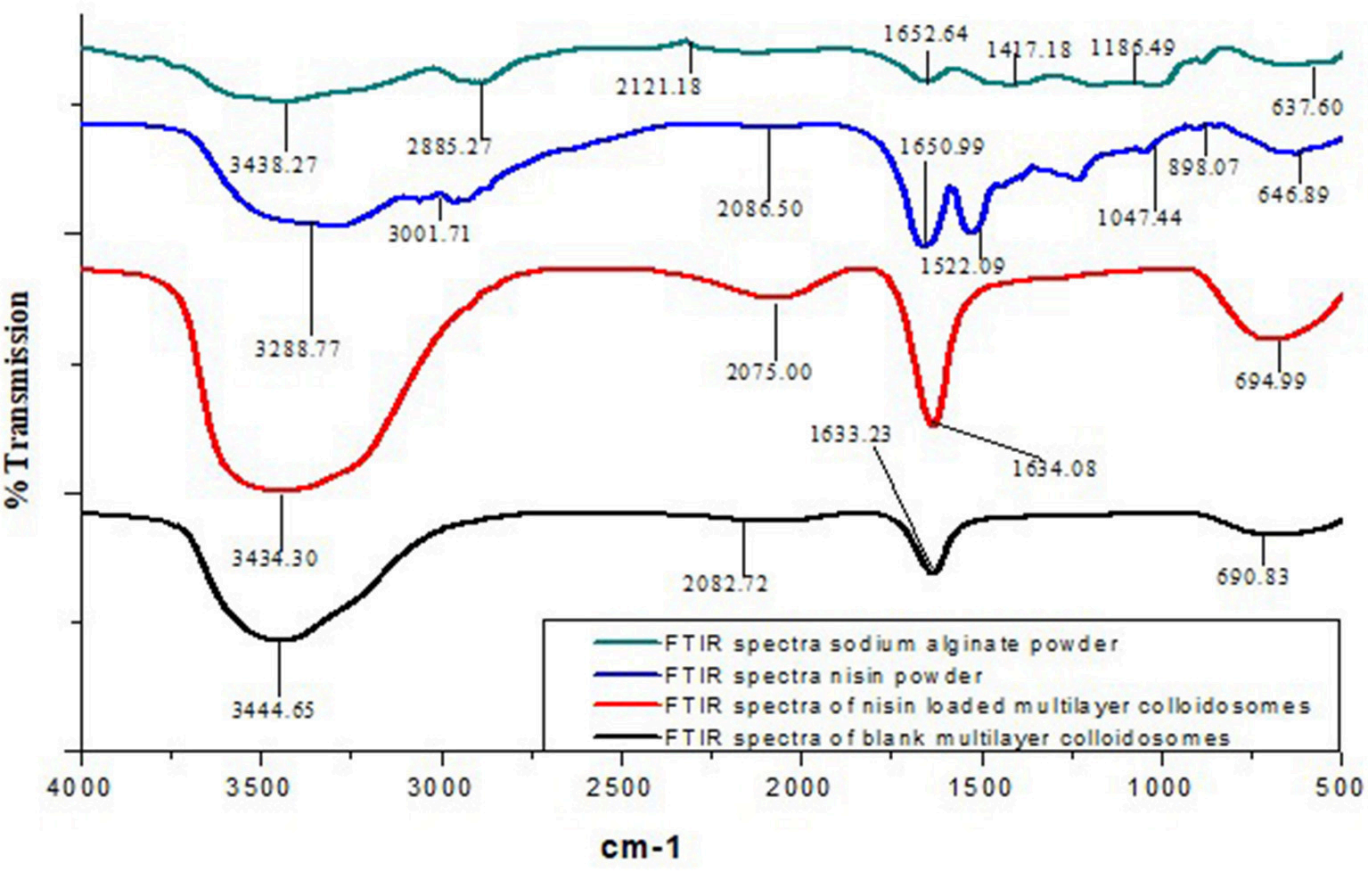
Comparative FTIR analysis of multicomponent NCS, pure powdered form of sodium alginate (outer layer), nisin powder and nisin loaded MCCS.

Figure 6 illustrates the FTIR spectra for nisin, alginate (surface layer) and multi-component colloidosomes (MCCS) as an entire carrier system. In FTIR spectral analysis of nisin powder, four major characteristics peaks appeared at 3,288, 3,001, 1,650, and 1,522 cm^−1^; which were attributed to O-H asymmetrical stretch, C-H symmetrical stretching, amide group, and to the bending of primary amines respectively. Sodium alginate displayed major characteristic peaks of O–H stretching at 3,438 cm^−1^, C-H stretching of the CH_2_ groups at 2,121 cm^−1^, carboxylate salt group (asymmetric stretching) at 1,652 cm^−1^, carboxylate salt group (week symmetric stretching) at 1,417, C-H stretching peak at 2,885 and C–O–C stretching at 1,186 cm^−1^. As described previously by Niaz et al., four major characteristic peaks at 3,398, 2,878, 1,654, and 1,379 cm^−1^ are due to the N–H, C–H, amide I and amide III groups respectively in the chitosan FTIR spectra (Niaz et al., 2016). In case of blank multicomponent colloidosomes, widening of carboxylate stretching peak with slightly shifted to lower wave numbers, which could possibly be the result of cross-linking between ALG and chitosan polymers. Furthermore, all characteristic peaks present in blank MCCS are also present in nisin loaded multicomponent systems. But all these spectral peaks shifted to lower wavenumber, which might be due to gelatinization property of alginate. Furthermore, no new peak appeared in nisin loaded MCCS, thus confirming that there is no chemical interaction between encapsulated nisin and alginate polymer. This observation supports the concept that nisin was not attached on the surface but encapsulated inside the polymer layer or inner core.

### *In vitro* antimicrobial activity of nano-antimicrobials

**Figure 7:**
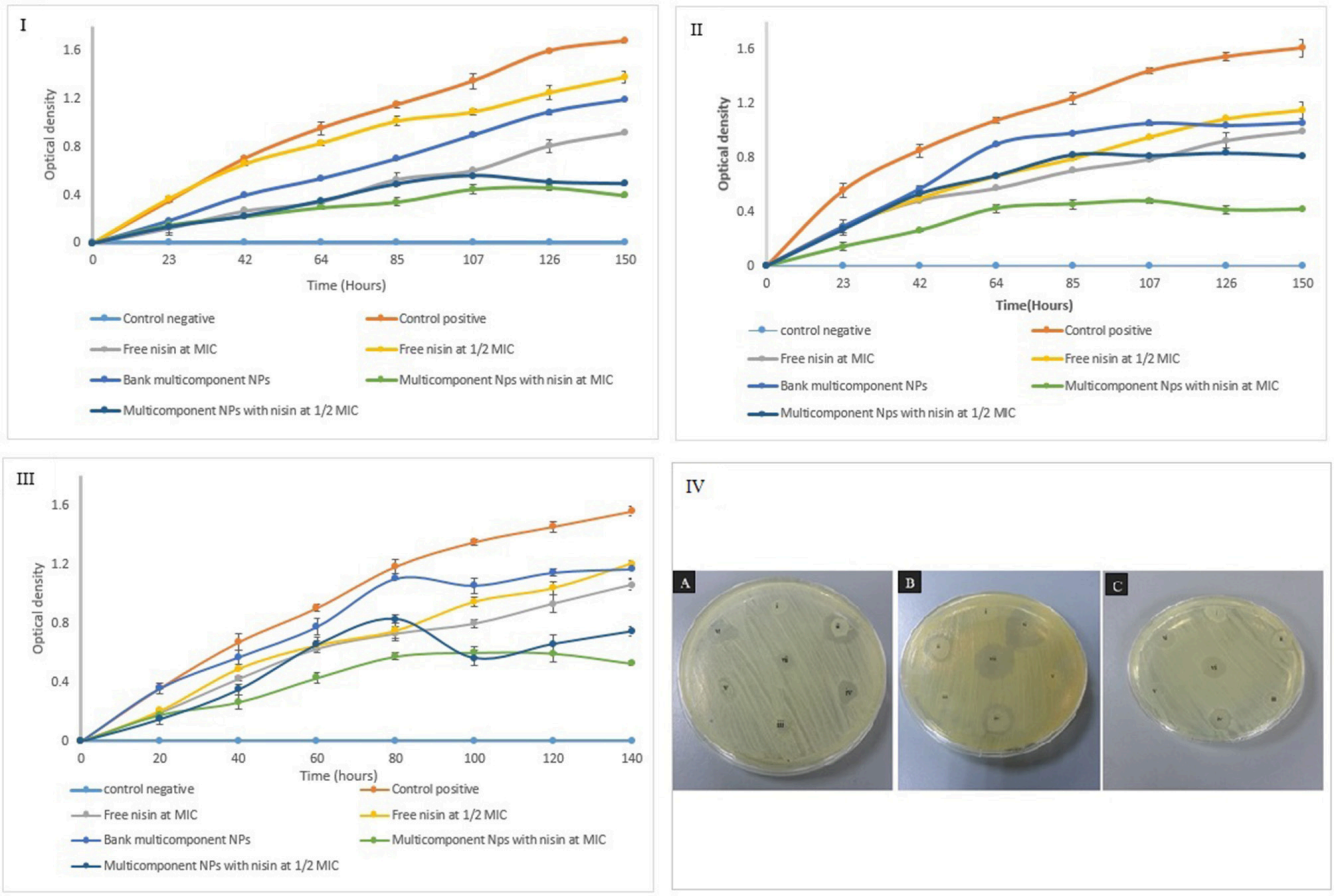
Growth kinetics of *Staphylococcus aureus* **(I)**, *Listeria monocytogenes* **(II)**, and *Enterococcus faecalis* **(III)** incubated with free nisin at MIC, ½ MIC and encapsulated nisin at MIC, ½ MIC in multicomponent nano-antimicrobial system **(IV)** antimicrobial potential of nano- antimicrobials against **(A)** *E. faecalis*, **(B)** *L. monocytogenes*, and **(C)** *S. aureus*. Antimicrobial activity of (i) blank liposome (ii) nisin encapsulated liposome (iii) blank chitosomes (iv) nisin loaded chitosomes (v) multicomponent alginate coated chitosomes (vi) nisin loaded multicomponent NCS (vii) free nisin.

Efficacy of different nisin loaded nano-carrier systems and their controls against selected foodborne pathogens (*Staphylococcus aureus, Listeria monocytogene*s, and *Enterococcus faecalis)* was tested on inoculated agar medium (Figures 7IVA–C). As predicted, void liposomes had not demonstrated any inhibition zone. However, in case of CTS coating on nano-liposomes (chitosomes) and alginate coated chitosomes (MCCS); small inhibition zones were observed owing to antibacterial activity of chitosan polymer. Active carrier-systems containing antimicrobial peptide nisin had generated larger inhibition zones due to release and diffusion of nisin in the agar network from colloidal systems. Active formulations of liposomes, chitosomes and multicomponent colloidosomes had exhibited better control of pathogen as compared to controls.

### Growth kinetics of resistant pathogens: potential of nano-antimicrobials

Afterwards, growth kinetics of selected pathogens were carried out under the influence of either free nisin or encapsulated in multi-component colloidosomes. Different solutions including control (without nisin addition), free nisin solution (MIC and ½ MIC), encapsulated nisin in MCCS at corresponding MIC and ½ MIC value were tested for antimicrobial activity against above-mentioned foodborne pathogens. As expected, the control sample without nisin had not shown antimicrobial action against all three resistant pathogens. Free nisin (MIC value and ½ MIC) inhibited bacterial growth up to 24 h but afterwards gradual and steady growth of bacteria was observed. However, the reduction of nisin concentration to ½ MIC did not control the increase in bacterial population as much as ½ MIC concentration of nisin in nano-encapsulated form.

Against S*taphylococcus aureus*, active multilayer nano-antimicrobials demonstrated better activity at ½ MIC concentration than free nisin at full MIC (Figure 7I). This reduction in pathogen growth was due to slow release of nisin initially from alginate-chitosan layer, secondly from chitosan-liposomes layer after swelling of alginate layer in the medium (Lopes et al., 2016) and then burst release after 5th day from liposome core.

Antibacterial activity of formulated NCS were also tested against *L. monocytogenes* (Figure 7II). It was observed that multicomponent NCS loaded with nisin at ½ MIC (25 μg/mL) concentration as well as void NCS controlled the bacterial growth similar to free nisin at identical concentration. Due to gelling property of ALG, bacterial growth was hindered even by blank MCCS. Similar observations have also been reported in the previous study, showing slower growth rate and narrower growth boundaries for *L. monocytogenes* in gelling agent e.g., sodium alginate and gelatin (Aspridou et al., 2014). However, an increase in bacterial growth was observed after 2 days due to alginate degradation in liquid medium, subsequently sustained growth was observed till 1 week. As expected, active multicomponent colloidosomes containing nisin at MIC controlled the bacterial growth almost double than free nisin at similar concentration. This could be attributed to the controlled and sustained release of bacteriocin from multilayered colloidosomes as well as synergistic antibacterial effect of CTS and nisin.

Activity of MCCS with and without active agent against *E. faecalis* was tested (Figure 7III) and it was observed that blank MCCS did not control bacterial growth during initial phase. After 42 h, slight decrease in bacterial growth was observed than positive control. Due to bacteriostatic, gelatinizing and antibacterial property of ALG, sol–gel transition occurred which stressed the microorganism and resulted in decreased growth rate (Szekalska et al., 2016). However, after 4 days empty multicomponent systems could control the bacterial growth as much as ½ MIC of free nisin because of exposed chitosan layer. Similar behavior was observed with ½ MIC (100 μg/mL) encapsulated nisin in multicomponent systems. Though after 4 days, progressive increase in swelling leads to disintegration of alginate coating, which exposes chitosan layer (Mukhopadhyay et al., 2016). Consequently, synergistic antibacterial effect of CTS and encapsulated nisin further reduced the bacterial growth.

Active MCCS containing nisin at MIC (200 μg/mL) controlled the growth of *E. faecalis* almost twice than free nisin at identical concentration due to sustained release of bacteriocin from multilayers system.

### Quantitative analysis of nano-antimicrobials' potential

**Figure 8:**
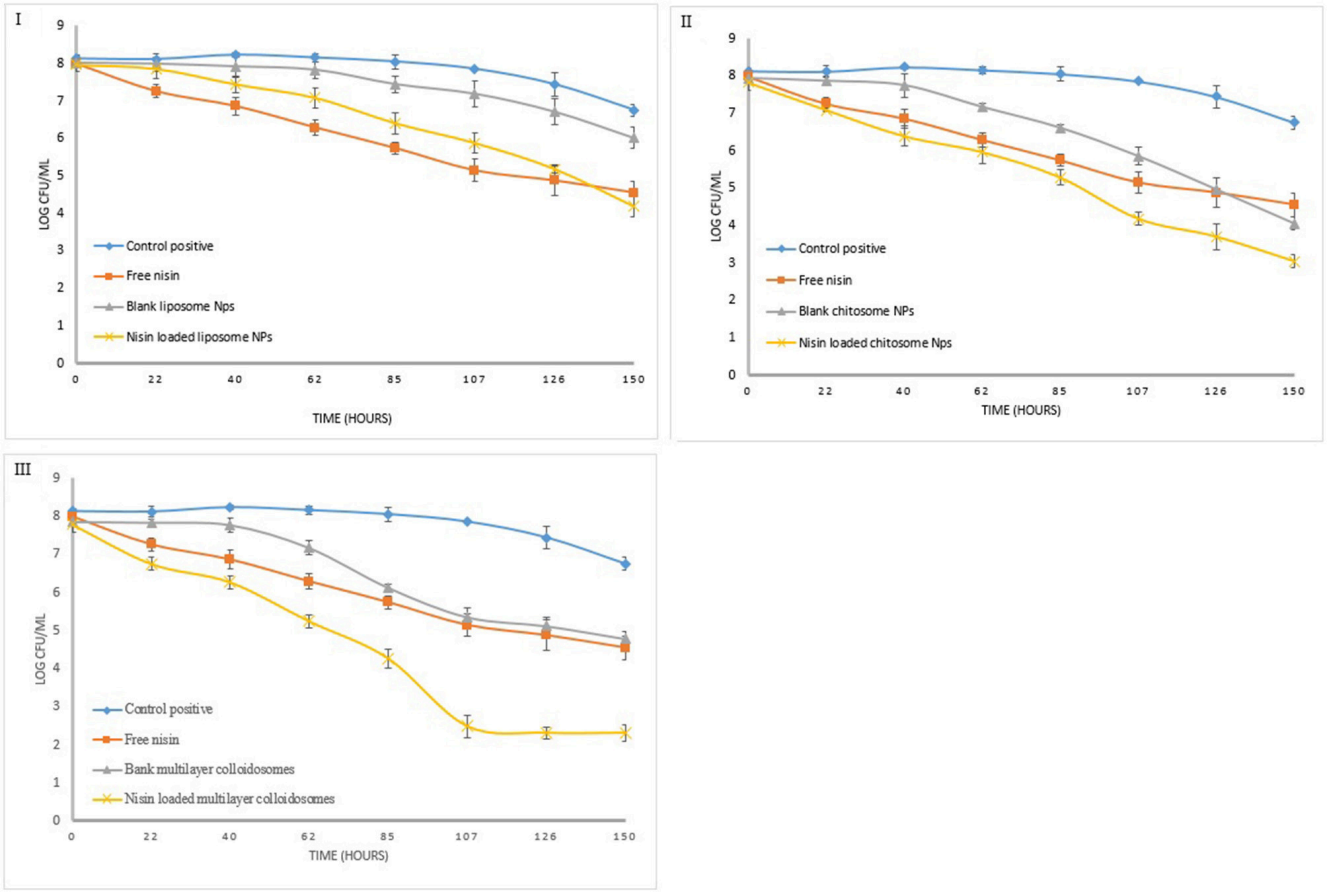
Quantitative antibacterial analysis of free nisin and encapsulated nisin in **(I)** liposomes, **(II)** chitosomes, and **(III)** multicomponent colloidal system against *L. monocytogenes*.

Figure 8 reveals the data observed during the quantitative antibacterial evaluation of *Listeria monocytogenes* by free nisin and various non-active and active nano-systems for 1 week. Positive control referred to bacterial growth in nutrient broth without any empty and active colloidal system. After 24 h of exposure, nanoliposome containing nisin did not reduce the viable counts (CFU mL^−1^) of *Listeria monocytogene*s as compared to free nisin which was able to reduce it by more than 1.5 log units.

Blank chitosomes decreased the bacterial count similar to free nisin after 1 week which is 3.4 log unit. However nisin loaded chitosomes reduce bacterial count by 4.8 log unit owing to controlled release of nisin from CTS coated liposomes, more encapsulation efficiency of chitosomes than liposomes and synergistic antimicrobial effect of chitosan and nisin (Hu et al., 2017).

Active multicomponent colloidosomes reduced the bacterial count by more than 1 log unit after 24 h. More than 2 log reductions in *Listeria* count was observed from 4th to 5th day, which might be due to burst release of nisin from multicomponent colloidosomes once alginate layer was degraded. Whereas, blank multicomponent nano-systems fail to control *Listeria* growth initially but once alginate layer disintegrated, exposed chitosan layer reduced the bacterial count for more than 2 log units.

**Figure 9:**
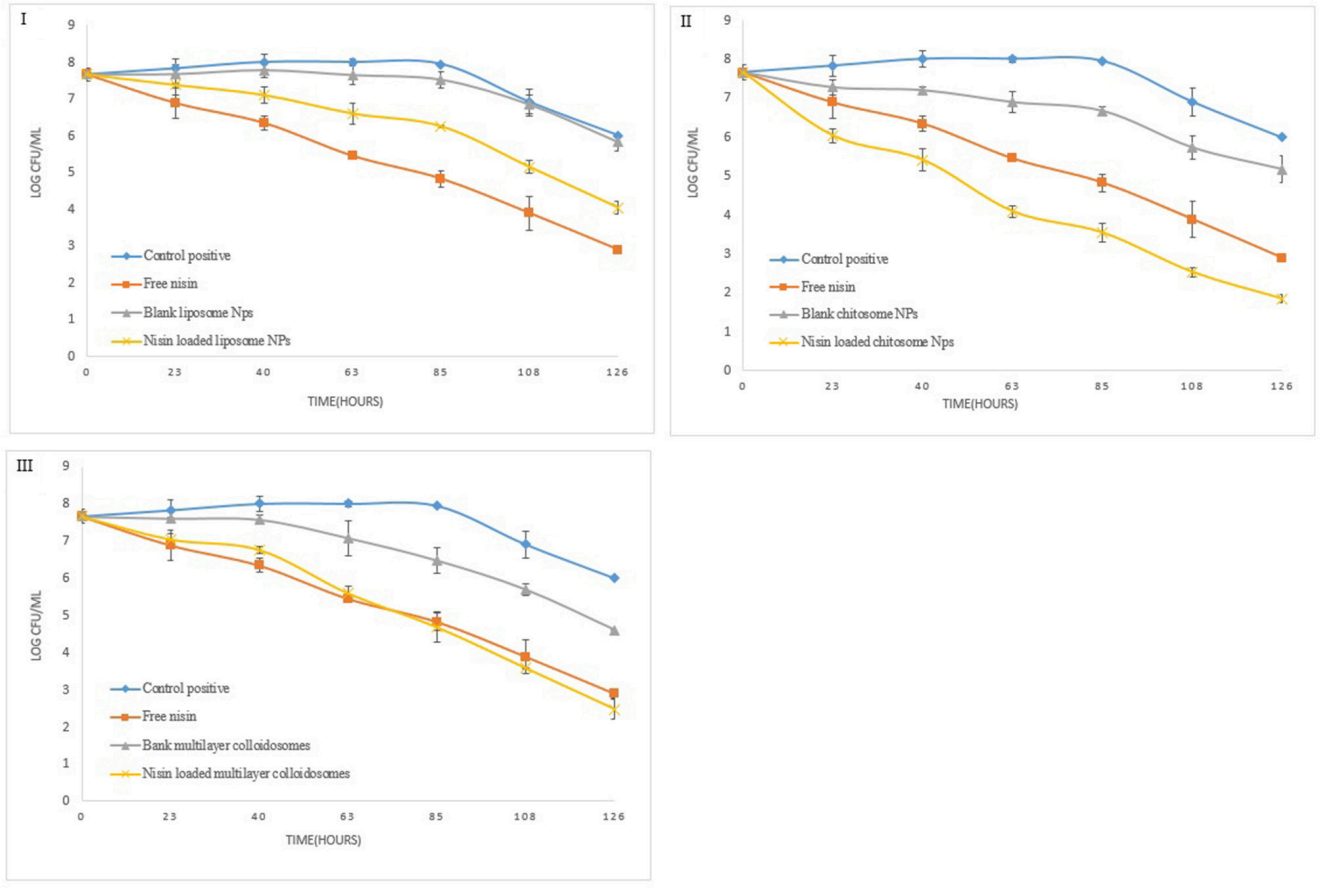
Quantitative antibacterial analysis of free and encapsulated nisin in **(I)** liposome, **(II)** chitosome, and **(III)** multicomponent colloidal system against *S. aureus*.

In quantitative antibacterial analysis for *S. aureus*, active liposomes did not exhibit good control as compared to free nisin at similar concentration (Figure 9). Total 1.5 log unit reduction in viable bacterial count was observed in first 5 days. Nevertheless, 2.5 log unit reduction in the bacterial count was observed in last 2 days due to controlled released of nisin, which is in coherence with the trend observed previously (Alishahi, 2014).

Chitosan coated liposomes demonstrated good control against *S. aureus* (Figure 9), as within 24 h almost 2 log unit reduction in the bacterial growth was observed. Though with free nisin, only 1-unit log reduction was observed. Continuous release of nisin and chitosan's antibacterial activity resulted in total 5.8 log unit reduction in the bacterial growth. Multicomponent NCS controlled the bacterial growth just like free nisin, however after 5th day relatively more reduction in pathogen count was observed than free nisin. These results correlate well with *in vitro* growth kinetics of *S. aureus* under the influence of nano-antimicrobials.

**Figure 10:**
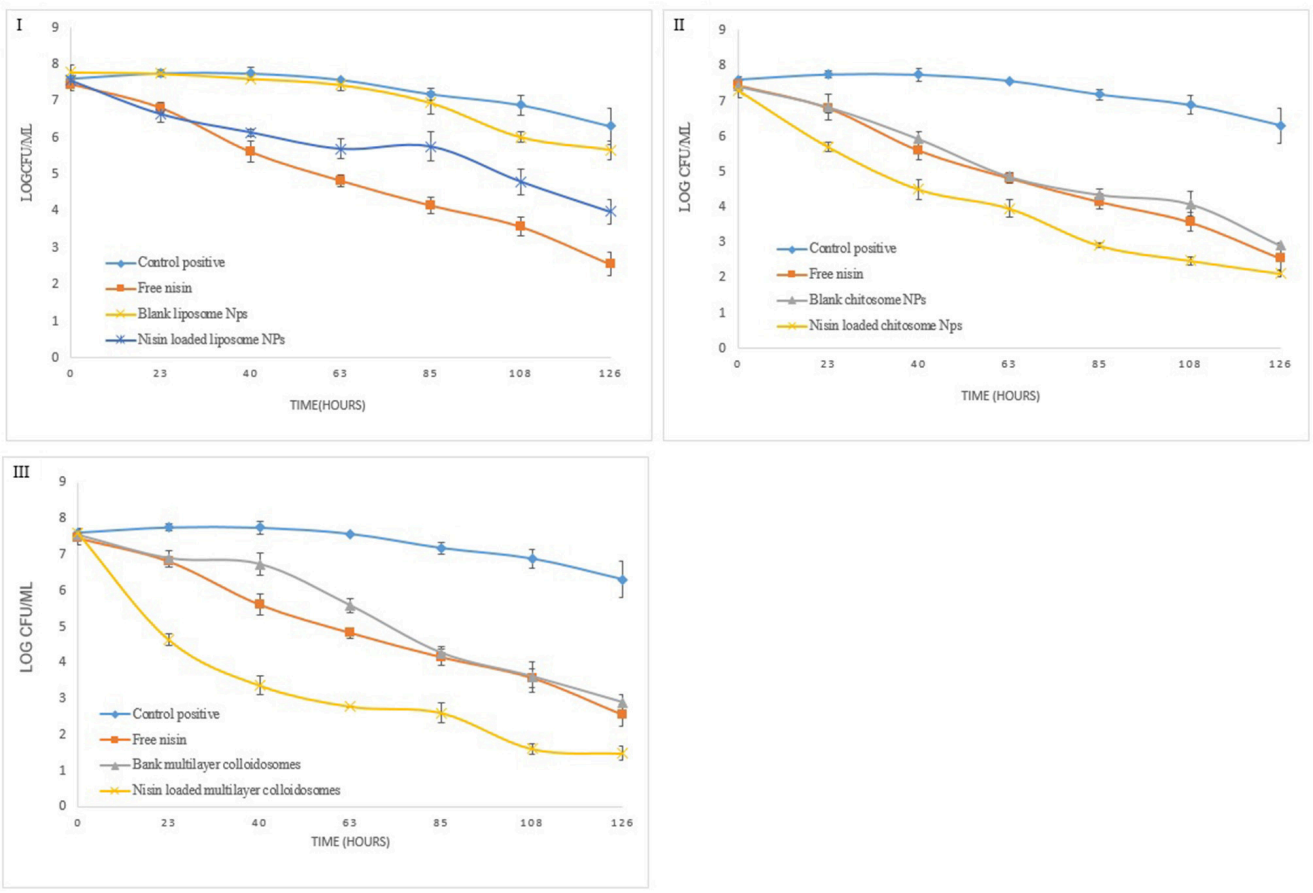
Quantitative antibacterial analysis of free and encapsulated nisin in **(I)** liposome, **(II)** chitosome, and **(III)** multicomponent colloidal system against *E. faecalis*.

Similarly, for *Enterococcus faecalis*, blank liposomes were not able to reduce bacterial count as shown in Figure 10. By using nisin loaded liposomes, 0.5 log unit reduction in viable bacterial cell count was observed in first 24 h (8). *Enterococcus* count further reduced to only 2 to 2.5 log units due to controlled release phenomenon. However, this reduction in bacterial count was less than free nisin. But for CTS coated liposomes, in first 24 h, 2 log unit reduction in bacterial count was observed which further continued to a total of 5.2 log unit decrease in 1 week.

Active multicomponent colloidosomes demonstrated best control over *Enterococcus faecalis*. Three log unit reduction was observed in 24 h. *E. faecalis* enumeration was further reduced to a difference of 2–2.5 log units after 72 h for active multicomponent systems. Total 6.1 log unit reduction in viable *Enterococcus* count was observed in 1 week.

Overall, we can conclude that the multicomponent colloidosomes exhibited an unmatched control against *L. monocytogenes* and *E. faecalis*. These resistant pathogens were not able to grow in the presence of multicomponent NCS due to two reasons, one by enhanced sustained release mechanism of nisin from multicomponent carriers (Fernandez-Saiz et al., 2010), and secondly alginate's gelling capacity reduced the bacterial growth by altering the oxygen and nutrients composition in the medium.

### *In vitro* controlled release kinetics of nisin from multicomponent NCS

**Figure 11:**
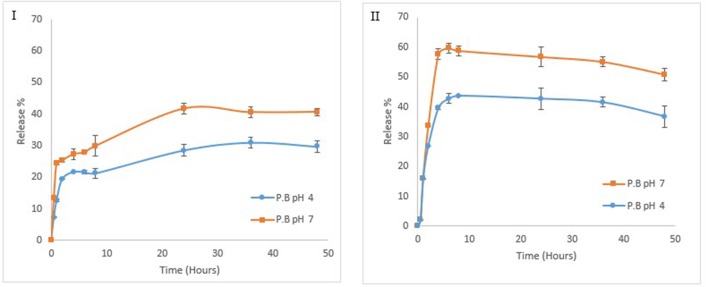
Effect of different temperatures and pH on release kinetics of nisin from formulated NCS at 4°C **(I)** at 37°C **(II)**.

To check the controlled release of nisin in phosphate buffer saline (PBS), an *in vitro* experiment was conducted at pH 7 and 4 on different temperatures e.g., physiological temperature (37°C) and storage refrigerated temperature (4°C). Sustained release of nisin from multicomponent NCS was monitored for 48 h (Figure 11). During initial hours, drug release rate increased gradually in similar manner at both temperatures and pH, but afterwards observed drug release rate was higher at 37°C as compared to 4°C from multi-component colloidosomes. At 4°C nisin release was observed to be 41 ± 1.6% in PBS at pH 7 and 28 ± 1.8% at pH 4 after 24 h. While at physiological temperature, 58 ± 1.5% and 43 ± 1% of the nisin released within 6 h at pH 7 and pH 4, respectively. Our observations indicated that there was a burst release during first 10 h at both pH values at 4°C. However, the amount released was higher at 37°C for both pH values than 4°C. Afterwards, the release rate was slow and steady in both pH conditions at different temperatures.

Whereas at 37°C, rapid nisin release were observed in first 6 h. Thus, a total of 59 ± 1.7% and 43 ± 3.6% of the nisin was released within 6 h at pH 7 and pH 4, respectively. After this point, gradual decrease in nisin release was observed at this temperature for both pH conditions. At low pH (slightly acidic pH), positively charged CTS and tight network of ALG coating help in retaining the nisin and it is expected that this will also help in protecting the encapsulated nisin against the proteolytic degradation in the food systems. On the contrary, at pH 7, relatively higher nisin release from multilayer colloidosomes could be realized due to the increased interaction between alkaline solvent and alginate shell. Moreover, high dissolution of alginate at this pH help in penetration of the solvent from alginate layer to chitosan layer in which nisin is present (Mukhopadhyay et al., 2015).

Furthermore, to investigate the mechanism of release kinetics (Fickian & non Fickian diffusion), Higuchi and Korsmeyer–Peppas mathematics models were applied to the data. Release profiles of nisin from MCCS were evaluated through regression coefficient (*r*^2^) and release exponent (n). As shown in Table 3, the *r*^2^ values at pH 4 for both temperatures (37°C and 4°C) were close to 1. However, regression coefficient at pH 7 on 37°C by Higuchi model was considerably lower than 1. This poor fitting data of nisin release at pH 7 indicated the change in surface area of MCCS as a function of temperature. According to our study, controlled release data of nisin from MCCS was best fitted by Korsmeyer–Peppas model. Release exponent (n) value for release of nisin at both temperatures and pH were less than 0.45. Therefore, Fickian diffusion is the controlling factor in nisin release from MCCS.

**Table 3:** *In Vitro* release kinetics parameters of nisin from multicomponent collidosomes (MCCS).

**Nisin loaded MCCS**		**Higuchi**	**Korsmeyer-peppas**	
**Temperature**	**pH**	**Correlation coefficient *r*^2^**	**Correlation coefficient *r*^2^**	***n***
37°C	pH 4	0.9557	0.9697	0.36 ± 0.01
	pH 7	0.8632	0.9758	0.24 ± 0.11
4°C	pH 4	0.9226	0.9914	0.41 ± 0.02
	pH 7	0.9147	0.9002	0.21 ± 0.05

### *In vitro* hemolysis assay

**Figure 12:**
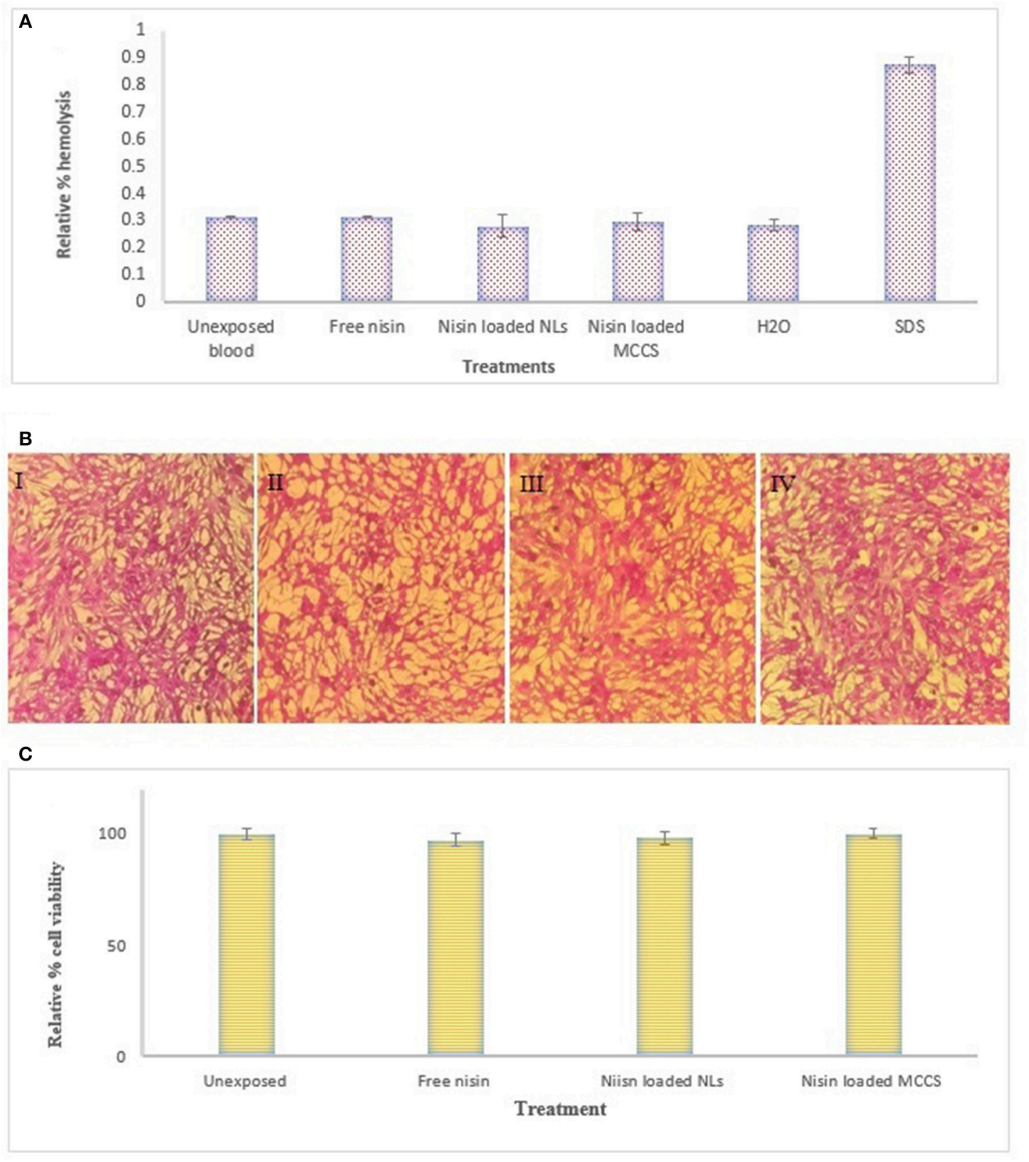
Cytotoxicity analysis of nano formulations. **(A)** *In vitro* hemolysis assay on human blood cells. Evaluation of different formulation e.g., nisin, nisin loaded NLs and nisin loaded MCCS for relative % hemolysis, **(B)** Cytotoxicity analysis on HepG2 liver cells treated with **(I)** untreated HepG2 cells **(II)** nisin **(III)** nisin loaded liposomes **(IV)** nisin loaded multicomponent colloidal system, **(C)** Relative % cell viability of different nano-formulations against HepG2 determined by using SRB; following treatment with free nisin, nisin loaded liposomes and nisin loaded MCCS. All assay values were taken in triplicate and mean values ± SD were calculated.

*In vivo* cytotoxic behavior of multicomponent colloidosomes could be predicted by *in vitro* hemolysis assay. As a potential antimicrobial food preservative, MCCS may pass through epithelial membrane and come in contact with blood cells, therefore it is essential to analyze their hemolytic effect. Hence the prepared NLs and MCCS were tested for hemocompatibility (Figure 12A). All the nano-antimicrobials used in this study have shown negligible hemolysis relative to the value obtained for control positive (1% SDS).

### *In vitro* cytotoxicity and cell viability studies on HepG2 cell lines

Cytotoxicity tests for different nano-formulations were performed on HepG2 cell lines (Figures 12BI–IV). The cytotoxicity of all the nano-formulations was negligible. For further cytotoxic analysis, SRB assay was performed to analyze the effect of MCCS on density of hepatic cells (Figure 12C). After 24 h, hepatic cells monolayer was not affected by nisin loaded MCCS, and the cell density remained like the untreated control. These findings are in coherence with previous results reported for non-toxicity of chitosan based nano-antibiotics (Jamil et al., 2016a).

## Conclusion

Carbohydrate polymers provide a useful platform to improve stability, entrapment, and controlled release of a wide variety of active agents. This study examined the influence of sodium alginate and CTS polymers' coating on the stability of liposomes. Physical properties of the fabricated multicomponent NCS revealed that these multilayer systems have high encapsulation efficiency than uncoated liposomes. Adding multiple layers and cross-linking of polymers on nano-liposome also improved their thermal stability without affecting their morphology. Multicomponent systems provided sustained release of antimicrobial agent (nisin) over longer period by Fickian diffusion, while being hemocompatible and nontoxic to human cells. Thus, due to enhanced controlled release and stability of nisin, these multi-component colloidosomes could be useful for incorporating antimicrobial agents into functional foods e.g., dairy products and beverages to combat multidrug resistant pathogens in food products.

## Author contributions

TasN carried out all of the research work, compiled data and wrote the manuscript. SS performed TGA and DSC analyses and TayN helped in FTIR based studies of interaction between drug and polymer. RA performed the cytotoxicity assays. ZR carried out the zeta-sizer analyses. MI provided scientific and technical assistance in experimentation and compilation of data and manuscript write up.

### Conflict of interest statement

The authors declare that the research was conducted in the absence of any commercial or financial relationships that could be construed as a potential conflict of interest.
